# The association between pelvic asymmetry and non-specific chronic low back pain as assessed by the global postural system

**DOI:** 10.1186/s12891-020-03617-3

**Published:** 2020-09-05

**Authors:** Qiuhua Yu, Huanjie Huang, Zhou Zhang, Xiaoqian Hu, Wenfeng Li, Le Li, Min Chen, Zhenwen Liang, Wai Leung Ambrose Lo, Chuhuai Wang

**Affiliations:** 1 Department of Rehabilitation Medicine, The First Affiliated Hospital, Sun Yat-sen University, 58 Zhong Shan Er Lu, Guangzhou, 5100800 China; 2 School of Biomedical Sciences, Li Ka Shing Faculty of Medicine, The University of Hong Kong, Hong Kong, China; 3 Guangdong Engineering and Technology Research Centre for Rehabilitation Medicine and Translation, The First Affiliated Hospital, Sun Yat-sen University, Guangzhou, China; 4 Department of Rehabilitation Medicine, The First Affiliated Hospital of Jinan University, Guangzhou, China

**Keywords:** Pelvic asymmetry, Low back pain, Photographic assessment, Pelvic posture

## Abstract

**Background:**

Empirical evidence that demonstrates the relationship between pelvic asymmetry and non-specific chronic low back pain (NCLBP) is currently lacking.

**Objective:**

To establish the reliability of the Global Postural System (GPS) in assessing pelvic asymmetry and identify the association between pelvic asymmetry parameters and the occurrence of NCLBP in young adults.

**Design:**

A cross-sectional, regression study.

**Methods:**

People who were aged between 18 and 30 and were diagnosed with NCLBP were recruited. Healthy individuals who were matched for age, sex, and education level were recruited as controls. Global Postural System (GPS) was employed to assess pelvic asymmetry. Prior to exploring the association, the reliability of GPS was assessed by the ICC (2, k) for interrater reliability, ICC (3, k) for intra-rater reliability, standard error and minimal detectable difference. Bivariate correlation analysis and logistic regression analysis were used to determine the relationship between pelvic asymmetry and the occurrence of NCLBP.

**Results:**

Twenty-eight healthy participants and 28 people with NCLBP were recruited. Moderate to excellent ICCs were observed for the inter-rater and intra-rater reliability of most postural parameters. The bivariate correlation analysis indicated that age, body mass index and pelvic asymmetry parameters were related to the occurrence of NCLBP. Pelvic angle asymmetry (odds ratio = 1.17), and asymmetry of the distance between the posterior superior iliac spine and the floor (odds ratio = 1.21) were associated with NCLBP.

**Limitations:**

This study did not explore the causal relationship between pelvic asymmetry in the sagittal plane/pelvic asymmetry in the transverse plane and the occurrence of NCLBP. The interpretation of the results may not be generalized beyond the sample population.

**Conclusions:**

The GPS is a reliable method to assess pelvic asymmetry in a clinical setting. Two pelvic parameters were associated with the presence of NLBP. Measurement of pelvic asymmetry may assist in the early identification of potential occurrence of NCLBP but further work is required.

## Introduction

Low back pain (LBP) creates a substantial socioeconomic burden for individuals worldwide [1]. The prevalence of LBP is approximately 14% and is increasing in the young adult population [1]. Most people who experience LBP do not have a recognisable or specific pathology, such as nerve root compression or serious spinal pathology [2]. Thus, this pathological condition is often referred to as nonspecific LBP [2, 3]. Pelvic asymmetry has been reported to be a potential contributor to the development of LBP and a primary source of pain [4, 5]. To date, there is a lack of empirical evidence to demonstrate if pelvic asymmetry is associated with LBP in the young adult population. Identifying the relationship between pelvic asymmetry and LBP may assist in the early identification of chronic LBP.

Pelvic asymmetry refers to the asymmetrical alignment of the pelvic bone in the frontal plane (lateral pelvic tilt), sagittal plane (iliac anterior/posterior rotation asymmetry) [6], or transverse plane (pelvic axial rotation) [7, 8] relative to the vertical axis. The classic overload principle suggests that anatomic adaptation of biological tissues occur when tissues are stressed beyond the normal stress level during tasks of daily living [9]. A large number of studies have demonstrated asymmetrical tissue adaptations in bone and muscle girth in people who participate in sports that are predominantly unilateral [10–12]. Therefore, directional asymmetry can be interpreted as the asymmetrically anatomic adaptations that occur in response to repetitive unilateral biomechanical loading [13, 14]. The anatomical asymmetry of the pelvis, which was similar to that of lower limbs, was also associated with the directional biomechanical loading [13]. It has been suggested that pelvic asymmetry may be related to the development of nonspecific chronic LBP (NCLBP), since lateral pelvic tilt is highly related to asymmetrical lumbar movement and places abnormal mechanical stresses on the body [15], which increases the strain on the soft tissues in the lumbar region [16]. The abnormal stresses on the soft tissues may subsequently contribute to the development of LBP [17].

In the last decade, several methods have been developed to perform accurate postural evaluations during standing. Technological advancements have enabled the use of highly reliable and easy-to-operate tools, such as X-ray scanners [18, 19] and computerized photographic systems [20], to assess postural asymmetry. X-ray imaging is the standard method of assessing spinal alignment because it provides clear images of anatomical landmarks. However, X-ray imaging is not preferred in clinical and research settings for routine procedures because X-ray imaging is associated with radiation emission. The use of computerized photographic systems that assess posture by the positions of anatomical landmarks is the recommended approach [20] because they are simple, noninvasive, affordable and free of radiation [20, 21].

Acceptable levels of reliability [20, 22] and validity [23] of the photographic assessment systems have been reported in previous studies. To date, there is a wide variety of systems that are available for assessments with different levels of reported reliability. Published studies assessing pelvic and lower extremity alignments mostly investigated intra-rater reliability but not interrater reliability [21, 24, 25]. The Global Postural System (GPS) is a recently developed computerized photographic postural assessment system. The GPS hardware that comprises a digital camera, a frame with a ruler and a fixed platform that enables consistent landmark identification is advantageous compared to a traditional photographic system that comprises a single camera placed in front of the person being assessed. The reliability of the GPS to assess pelvic asymmetry is not available, and it is essential to establish its reliability before it is recommended for clinical use.

The first part of the study aimed to establish the reliability of the GPS to assess pelvic asymmetry in young adults in a clinical setting. The second part of the study primarily aimed to explore the association between pelvic asymmetry parameters and NCLBP occurrences in young adults by using a binary logistic regression model. It was hypothesized that the GPS was sufficiently reliable to assess pelvic postural asymmetry and that pelvic postural asymmetry was related to the occurrence of NCLBP.

## Methods

### Sample population

Participants were recruited from the staff and student populations of Sun Yat-sen University. The inclusion criteria for the NCLBP group were as follows: 1) age between 18 and 30; 2) diagnosis of NCLBP lasting more than 3 months; 3) pain score greater than 2 on the numerical rating scale (NRS) in both static (i.e., lying, sitting, or standing) and dynamic situations (i.e., moving or walking) was defined as the presence of LBP; 4) no referred symptoms of radiating pain below the knee, or paresthesia during straight-leg raise test [26]; and 5) no radiographic evidence of congenital anomalies of the lumbosacral region. The exclusion criteria for the NCLBP group were as follows: 1) presence of scoliosis as assessed by the Adam’s forward bend test [27]; 2) history of fracture or surgery in the pelvic or spinal area; 3) history of a neurological disorder or on regular medications; 4) Montreal cognitive assessment (MoCA) score of < 26;and 5) pregnancy. Healthy individuals who were matched for age, sex, and education level were recruited as controls. This study included participants with a minimum NRS score of 2 because part of the study aimed to obtain clinical information to potentially facilitate the early identification of low back pain. Thus, a slightly lower pain score was adopted as inclusion criteria. It was also for pragmatic reason to increase the sample size and enhance the statistical power.

### Ethics

Ethical approval of this study was obtained from the First Affiliated Hospital at Sun Yat-sen University (ETHICS No.[2019]206). An information sheet was provided to all participants prior to enrolment of the study. Written informed consent was obtained from all of the participants.

### Study settings and instruments

**Fig. 1:**
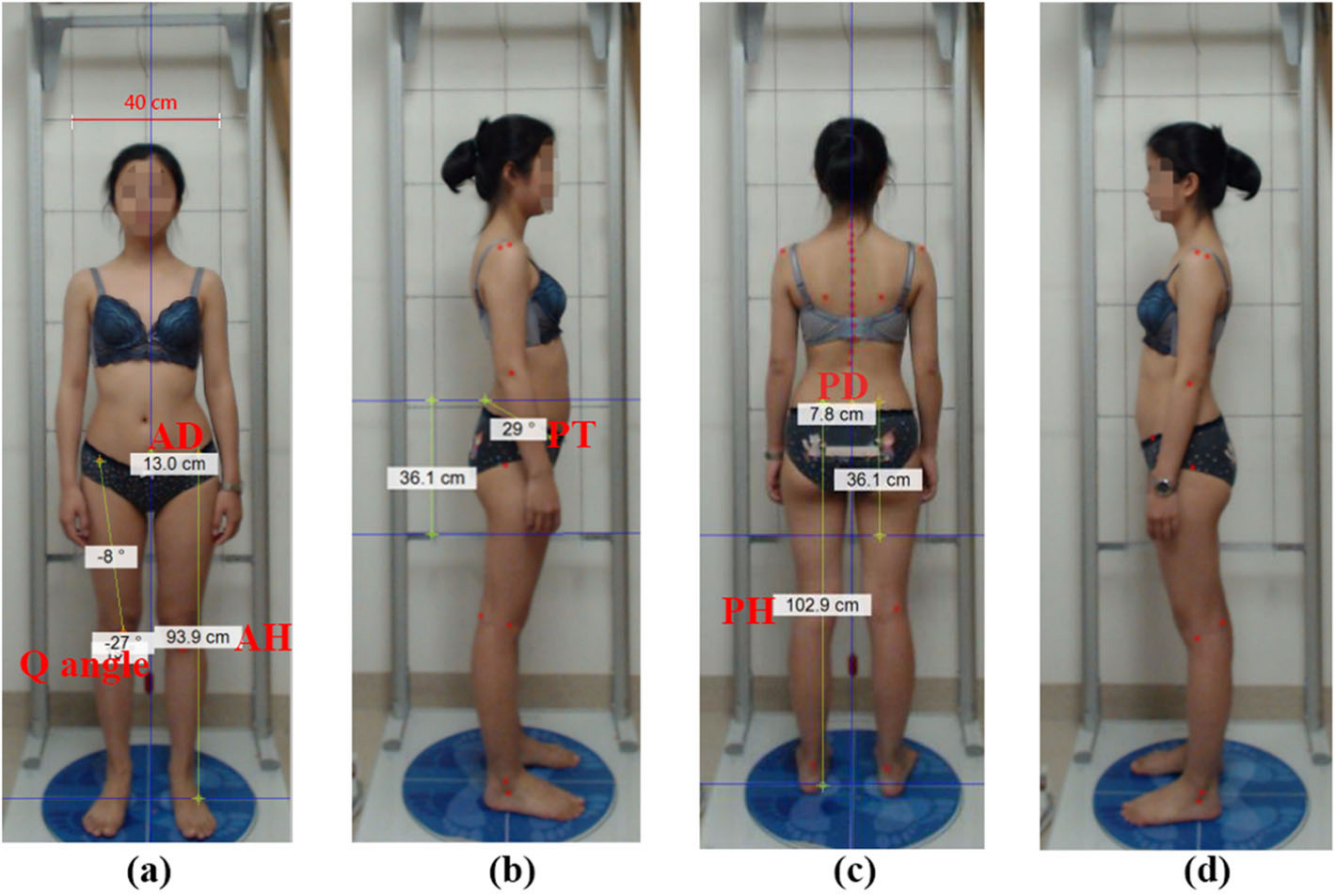
Photos with different views taken by the GPS. **a** front view; **b** right lateral view; **c** back view; **d** left lateral view. Keys: AD - the distance between ASIS and the midline; AH - the height of the ASIS from the platform; PT- anterior pelvic tilt angle; PD - the distance between PSIS and the midline; PH - the height of the ASIS from the platform

The present study was conducted in the postural examination room in the Rehabilitation Outpatient Department of the First Affiliated Hospital of Sun Yat-sen University. The bespoke GPS 5.0 software was adopted for photo acquisition and postural analysis. The GPS hardware comprised of two aluminium, vertical bars with rulers on the sides, a plumb line for postural reference, and an adjustable mirror on the top that was attached to a stable platform (Fig. 1). Two reference lines and four footprints facing different directions were used to calibrate the platform and enable consistent positioning of the feet. To set the scale of the lines, the horizontal distance between two vertical lines on the frame with rulers was recorded as 40 cm. A 1-m-tall digital camera with 2 megapixels (Logitech Pro C920; Logitech, China) was positioned 2.5 m away from the participant. The participant stood barefoot on the platform in undergarments while his or her posture was captured by the digital camera.

### Data collection procedure

Prior to commencing postural assessment, participants’ characteristics, of age, sex, education level, medication status, history of NCLBP in the past year, history of any other disease, and time spent on general physical exercise per week, were recorded in a demographic information sheet. The centres of the anatomical landmarks were first marked by the assessors using red stickers. The selected anatomical landmarks were the bilateral anterior superior iliac spine (ASIS), posterior superior iliac spine (PSIS), greater trochanter, tuberositas tibiae and midpoint of the patella. Postures were captured from the anterior, posterior and left/right lateral views (Fig. 1). During the postural examination, participants were asked to keep their usual body posture with their eyes looking straight ahead.

#### Reliability

For within-day inter-rater reliability of the GPS, healthy participants received a postural assessment by two different testers separately (tester A and tester B) during the first visit. Each tester was required to identify the anatomical landmarks and apply the red sticker at the centre of each landmark. The sequence of assessments by the two testers was randomized. Tester A then repeated the postural assessment procedure 1 week later to establish the intra-rater reliability. The two testers were trained by an experienced therapist who was not directly involved in the study. The two testers were not aware of the group allocation of each participant.

#### Pelvic postural assessment

Participants in the NCLBP group received the GPS assessment by tester A on one occasion. The pelvic postural parameters that were included in the data analysis were: 1) the left/right anterior pelvic tilt angle; 2) the left/right distance between the ASIS and the midline; 3) the left/right height of the ASIS from the platform; and 4) the left/right Q angle (see Fig. 1). The photo analyser module of GPS 5.0 software required the anatomical landmark to be manually confirmed from the recorded image by the assessor in order to calculate the parameter. This procedure was repeated 3 times to obtain the mean of each parameter. The mean values were used for data analysis and the calculation of pelvic asymmetry ratios. Similar to the methods used by Gnat and Bialy [8], the pelvic asymmetry ratios of each parameter were calculated first by dividing the parameter of the left side by the parameter of the right side to obtain a relative ratio between the two sides. Then, 1 was subtracted from this ratio to normalize the ratio. The equation that was used to quantify pelvic asymmetry is as follows:

$$ \mathrm{Asymmetry}\ \mathrm{ratio}\ \left(\%\right)=\mid \left( left\ pelvic\ postural\ parameter/ right\ pelvic\ postural\ parameter\right)-1\mid \mathrm{x}\ 100 $$

The pelvic asymmetry ratios that were calculated are: 1) the Q angle asymmetry ratio (QAR); 2) the height of the PSIS from the platform asymmetry ratio (PHAR); 3) the distance between the PSIS and the midline asymmetry ratio (PDAR); 4) the height of the ASIS from the platform asymmetry ratio (AHAR); 5) the distance between the ASIS and the midline asymmetry ratio (ADAR); and 6) the pelvic tilt angle asymmetry ratio in the sagittal plane (PTAR).

### Data analysis

All statistical analyses were conducted in SPSS ver 20.0 software (IBM SPSS Inc. Chicago, IL, USA). Statistical significance was set at *p* < 0.050. The sample characteristics were analysed by descriptive statistics. For the reliability analysis, relative reliability was determined by the intraclass correlation coefficient (ICC). As repeated measurements were recorded for each parameter, this study employed the ICC(2,k) model for the inter-rater reliability and ICC(3,k) model for the intra-rater reliability. ICC levels were interpreted as follows: Excellent: > 0.75; Good to Fair: 0.74–0.40; and Poor: < 0.40 [28]. Absolute reliability was determined by the standard error of measurement (SEM) and minimal detectable difference (MDD_95_) [29]. The MDD_95_ corresponds to the upper bound of the random variation that 95% of stable patients generate when tested on multiple occasions [30]. The formula for MDD_95_ is MDD = 1.96*SEM* $\sqrt{2\kern0.5em }$[30]. The pelvic asymmetry ratios are the ratio of pelvic postural parameters between left and right sides, which are affected by the measured values of the pelvic postural parameters.

To identify the relationship between pelvic asymmetry and NCLBP, the differences in the demographic variables (including age, height, weight, BMI, duration of exercise per week), pelvic postural parameters and pelvic asymmetry parameters between groups were tested using an independent *t*-test. A stepwise logistic regression analysis with a forward conditional method was employed to explore the association between pelvic asymmetry and NCLBP. Before testing this model, a bivariate Pearson correlation was performed to test the relationships between age, BMI, height, weight and the pelvic asymmetry parameters. The relationships between the occurrence of NCLBP and the other variables (including the BMI, height, weight and pelvic asymmetry parameters) were explored by Spearman correlation with the occurrences of NCLBP as ordinal data (the participants with NCLBP were marked as 1, and the controls were marked as 0). Adapting the methods used in the previous study [31], the age, BMI, height, weight, and pelvic asymmetry parameters were used as the independent variables in the logistic regression analysis.

## Results

### Participants

A total of 56 participants were recruited. Twenty-eight healthy participants (13 females and 15 males) who never experienced low back pain were recruited for the reliability analysis and as controls. Twenty-eight (14 females and 14 males) participants who reported experiencing chronic low back pain were included in the NCLBP group. The sample characteristics of both cohorts are presented in Table 1. Both groups were matched for age, height, weight and education levels with normal or corrected-to-normal visual acuity. No participants had a history of a neurological disorder, mental disorder or regular medication. Participants in the NCLBP group had a statistically higher BMI than those in the control group (*p* = 0.03).

**Table 1:** The characteristics of the two groups of participants

	NCLBP (*n* = 28) Mean (SD)	Control (*n* = 28) Mean (SD)	*t*, *p* value	95% CI
Age (years, Mean (SD))	22.21 (2.53)	22.61 (1.85)	*t* = 0.663, *p* = 0.51	−0.8,1.58
Height (cm, Mean (SD))	166.82 (7.98)	168.32 (8.46)	*t* = 0.682, *p* = 0.50	−2.91,5.91
Weight (kg, Mean (SD))	59.22 (9.35)	56.36 (10.51)	*t* = −1.076, *p* = 0.29	−8.19,2.47
BMI (kg/m^2, Mean (SD))	21.15 (2.16)	19.77 (2.51)	*t* = −2.219, *p* = 0.03	−2.64,-0.13
General exercise duration per week (hour, Mean (SD))	0.56 (0.52)	0.47 (0.49)	*t* = −0.662, *p* = 0.51	−0.36,0.18
NRS (static, Mean (SD))	2.85 (0.76)	–	–	
NRS (dynamic, Mean (SD))	3.82 (1.16)	–	–	

Keys: *BMI* Body mass index, *NRS* Numerical rating scale

### Reliability

Moderate to excellent ICCs were observed for within-day inter-rater and between-day intra-rater reliability of all parameters. Among all the parameters, the lowest ICC values were observed for the distance between the PSIS and the midline for both inter- and intra-rater reliability. Eight parameters were observed to have a lower bound of the confidence interval less than the acceptable lower bound of 0.75 for inter-rater reliability [32]. For intra-rater reliability, lower bound of the confidence interval for six parameters was less than 0.75; the ICC values, SEM and MDD_95_ for intra-rater reliability were higher than those for inter-rater reliability across all parameters. The SEM for all pelvic postural assessments ranged from 0.38 to 2.28 for inter-rater reliability and from 0.32 to 2.02 for intra-rater reliability. The MDD_95_ for all pelvic postural assessments ranged from 1.26 to 6.31 for inter-rater reliability and from 1.12 to 5.59 for intra-rater reliability. Table 2 presents the results of all reliability indices for inter- and intra-rater reliability of all the pelvic postural parameters.

**Table 2:** ICCs and absolute reliability for inter-rater and intra-rater reliability of all the pelvic postural variables

		Inter-rater reliability			Intra-rater reliability		
		ICC (95% CI)	SEM	MDD_95_	ICC (95% CI)	SEM	MDD_95_
Anterior pelvis tilt angle	Left	0.76 (0.48,0.89)	2.28	6.31	0.83(0.63,0.92)	2.02	5.59
	Right	0.77 (0.50,0.90)	2.15	5.95	0.78 (0.53,0.90)	2.00	5.55
Q angle	Left	0.78 (0.52,0.90)	1.83	5.06	0.89 (0.77,0.95)	1.48	4.10
	Right	0.74 (0.44,0.88)	1.82	5.04	0.79 (0.54,0.90)	1.80	4.98
The height of the ASIS from the platform	Left	0.96 (0.92,0.98)	1.35	3.74	0.99 (0.97,0.99)	0.79	2.20
	Right	0.97 (0.92,0.98)	1.27	3.52	0.98 (0.97,0.99)	0.85	2.37
The distance between the ASIS and the midline	Left	0.77 (0.50,0.89)	0.59	1.62	0.87 (0.72,0.94)	0.48	1.33
	Right	0.77 (0.24,0.91)	0.63	1.76	0.95 (0.89,0.98)	0.32	0.88
The height of the PSIS from the platform	Left	0.94 (0.88,0.97)	1.71	4.74	0.99 (0.98,0.99)	0.67	1.85
	Right	0.94 (0.87,0.97)	1.70	4.72	0.99 (0.98,0.99)	0.69	1.90
The distance between the PSIS and the midline	Left	0.72 (0.40,0.87)	0.38	1.07	0.73 (0.42,0.88)	0.45	1.26
	Right	0.68 (0.33,0.85)	0.46	1.26	0.71 (0.37,0.86)	0.40	1.12

### Pelvic postural parameters

The results of the descriptive statistics of the pelvic postural parameters for both groups are presented in Table 3. Independent *t*-tests revealed that between-group differences were not statistically significant in all pelvic postural parameters (*p* > 0.05), except for the distance from the left PSIS to the midline (*p* = 0.04). The between-group differences for the asymmetry ratios of the pelvic tilt angle in the sagittal plane (PTAR), the Q angle (QAR), the distance between the ASIS and the midline (ADAR), and the distance between the PSIS and the midline (PDAR) were significant (*p* < 0.05). The asymmetry ratios of the height of the ASIS from the midline (AHAR) and between the PSIS and the midline (PHAR) were not significant (*p* > 0.05).

**Table 3:** Means and standard deviations (SDs) of all pelvic postural variables and pelvic asymmetry parameters

	Mean (SD)						
		Control Group			NCLBP Group	*t*, *p value*	95% CI
		Tester A	Tester B: first time	Tester B: second time	Tester A		
Anterior pelvis tilt angle (^o^)	Left	21.04 (4.48)	21.04 (4.80)	21.36 (5.10)	22.29 (6.83)	*t* = −0.809, *p* = 0.42	−4.35,1.85
	Right	21.07 (4.99)	21.25 (4.00)	20.75 (4.53)	22.43 (7.06)	*t* = − 0.831, *p* = 0.41	−4.63,1.92
Q angle (^o^)	Left	20.00 (3.45)	21.00 (4.24)	21.21 (4.67)	19.64 (7.99)	*t* = 0.217, *p* = 0.83	−2.94,3.65
	Right	19.93 (3.24)	20.57 (3.87)	21.25 (3.89)	19.50 (7.97)	*t* = 0.263, *p* = 0.79	−2.83,3.69
The height of the ASIS from the platform (cm)	Left	103.16 (6.92)	102.76 (6.91)	102.70 (7.00)	100.11 (18.27)	*t* = 0.826, *p* = 0.41	−4.35,10.45
	Right	103.25 (6.81)	103.00 (6.76)	102.76 (6.75)	100.30 (17.96)	*t* = 0.812, *p* = 0.42	−4.33,10.23
The distance between the ASIS and the midline (cm)	Left	14.44 (1.09)	14.45 (1.35)	14.46 (1.33)	14.43 (1.55)	*t* = 0.030, *p* = 0.98	−0.71,0.73
	Right	14.73 (1.25)	15.54 (1.42)	15.45 (1.37)	14.76 (1.74)	*t* = −0.71, *p* = 0.94	−0.84,0.78
The height of the PSIS from the platform (cm)	Left	110.41 (7.51)	109.46 (6.96)	109.65 (6.41)	110.68 (6.88)	*t* = −0.141, *p* = 0.89	−4.13,3.59
	Right	110.56 (7.40)	109.41 (6.86)	109.43 (6.21)	110.66 (6.87)	*t* = −0.049, *p* = 0.96	−3.92,3.73
The distance between the PSIS and the midline (cm)	Left	5.52 (0.73)	5.68 (0.72)	5.79 (1.03)	6.28 (1.67)	*t* = −2.194, *p* = 0.04	−1.32,0.02
	Right	5.48 (0.83)	5.69 (0.79)	5.53 (0.72)	6.04 (1.29)	*t* = −1.948, *p* = 0.06	−1.14,0.02
PTAR (%)		8.3 (7.7)			19.9 (19.1)	*t* = −2.989, *p* = 0.01	− 19.5,-3.7
QAR (%)		10.3 (7.6)			51.6 (67.6)	*t* = −3.206, *p* = 0.003	−67.6,-14.9
AHAR (%)		0.7 (0.5)			1.1 (1.4)_	*t* = −1.416, *p* = 0.17	−0.9,0.2
ADAR (%)		3.7 (3.8)			7.5 (3.7)_	*t* = −3.825, *p* < 0.001	−5.8,-1.8
PHAR (%)		0.4 (0.3)			0.4 (0.4)	*t* = 0.679, *p* = 0.50	−0.1,0.2
PDAR (%)		6.3 (4.0)			21.8 (17.5)	*t* = −4.570, *p* < 0.001	−22.4,-8.6

Keys: *ASIS* Anterior superior iliac spine, *PSIS* Posterior superior iliac spine, *PTAR* Pelvic tilt angle asymmetry ratio in the sagittal plane, *QAR* Q angle asymmetry ratio, *AHAR* Height of the ASIS from the platform asymmetry ratio, *ADAR* Distance between the ASIS and the midline asymmetry ratio, *PHAR* height of the PSIS from the platform asymmetry ratio, *PDAR* Distance between the PSIS and the platform asymmetry ratio

### Logistic regression

The binary correlations between age, BMI, height, weight, pelvic asymmetry parameters and occurrence of low back pain are shown in Table 4. The results of the bivariate correlation indicated that age, BMI and several pelvic asymmetry parameters were related to the occurrence of NCLBP. In the regression model, only BMI (B = 0.48, *p* = 0.05, odds ratio (OR) = 1.62), PTAR (B = 0.15, *p* = 0.02, OR = 1.17), and PDAR (B = 0.19, *p =* 0.02, OR = 1.21) were found to be significant factors for NCLBP (Table 5). One person in the healthy group had larger PTAR than the mean PTAR in NCLBP group. Nine participants in the NCLBP group had smaller PTAR than the mean PTAR of the control group. For the PDAR, no participants in the control group had larger asymmetry than the mean PDAR of the NCLBP group. Five participants with NCLBP had lower PDAR than the mean PDAR of the NCLBP group. Other factors including age, height, weight, QAR, AHAR, ADAR, and PHAR were not significant factors.

**Table 4:** The binary correlations between age, BMI, pelvic asymmetry parameters and the occurrence of low back pain

		Age	Height	Weight	BMI	PTAR	QAR	AHAR	ADAR	PHAR	PDAR	Group
Age	Pearson	–										
Height	Pearson	.045	–									
Weight	Pearson	−.015	.745^**^	–								
BMI	Pearson	−.051	.271^*^	.840^**^	–							
PTAR	Pearson	.108	−.070	.011	.044	–						
QAR	Pearson	.074	.040	.246	.330^*^	.191	–					
AHAR	Pearson	−.211	.309*	.154	−.040	.300*	−.046	–				
ADAR	Pearson	−.022	.148	.271*	.271*	.284*	.238	.146	–			
PHAR	Pearson	−.026	−.287*	−.196	−.067	.196	−.037	−.073	.195	–		
PDAR	Pearson	−.081	−.033	.017	.051	.156	.137	.078	.353**	−.052	–	
Occurrence of NCLBP	Spearman^a^	−.286*	−.091	.114	.276*	.447**	.623**	−.001	.495**	−.125	.535**	–

*denotes *p* < 0.05; ** denotes *p* < 0.01; a denotes the relationships between the occurrence of NCLBP and other variables as explored by Spearman correlation

**Table 5:** Results of the stepwise logistic regression analysis with a forward selection method. *a* denotes that *p* is equal to *0.052*, which is marginally significant

Dependent variables	Independent variables	Non-stand partial regression coefficient	*p* value	OR	95% confidence interval for odds ratio	
					lower	upper
	BMI	0.48	0.05^a^	1.62	1.00	2.63
	PTAR	0.16	0.02	1.21	1.02	1.34
	PDAR	0.19	0.02	1.17	1.04	1.42

## Discussion

The current study investigated if the GPS was a reliable method for pelvic postural assessment when used in the clinical setting. It also explored the associations between pelvic asymmetry and the occurrence of NCLBP in the young adult population. The results of this study indicated that GPS was a reliable method for postural assessment. PTAR and PDAR were factors associated with NCLBP.

### Reliability

The results of the present study indicated moderate to excellent inter- and intra-rater reliability for distance variables surrounding the pelvis assessed by the GPS, except for the distance between the PSIS and the midline. These results were consistent with those reported in published studies [21, 24, 25]. A common issue related to the reliability of photographic pelvic posture assessments is the repositioning of markers between measurement sessions. The repositioning of markers at the centre of the ASIS and PSIS is particularly problematic due to the anatomical characteristics of these two points [21]. This study observed that the horizontal distances (distance between the ASIS and the midline; distance between the PSIS and the midline) had lower ICC values than the vertical distances (distance between the ASIS and the platform; distance between the PSIS and the platform). A potential reason for this observation is that the ICC values tend to be affected by the spread of the data [33]. The distances between the ASIS/PSIS to the midline are shorter than the distances between the ASIS/PSIS and the platform. Thus, the small range of the data may contribute to low ICC values. Parameters that involved the ASIS and PSIS also have higher MDD_95_ and SEM values for inter-rater reliability than for intra-rater reliability, suggesting more measurement errors occur in assessments between individuals than in assessments within individuals. Muscle contractions and excessive soft tissue surrounding the pelvis made it difficult to locate the PSIS and ASIS landmarks [21, 25]. This issue did not appear to be improved by thorough training prior to the data collections, which in theory should reduce the landmark repositioning error. The anatomical angle assessed by the GPS showed good to excellent inter- and intra-rater reliability. A previous study on intra-rater reliability reported an ICC of 0.84 and an SEM of 2.48 degrees and an ICC of 0.89 and an SEM of 2.16 degrees for pelvic tilt angle and Q angle, respectively [21]. For inter-rater reliability, a previous study reported an ICC of 0.43 and an SEM of 3.99 on the right and an ICC of 0.49 and an SEM of 4.24 degrees on the left for the pelvic tilt angle. An ICC of 0.84 and an SEM of 2.83 degrees on the right and an ICC of 0.89 and an SEM of 2.16 degrees on the left was reported for the Q angle [25]. The smaller SEM values observed in the present study compared with previous studies indicates that the GPS has less measurement error that previous methods when measuring angle parameters.

### The relationship between pelvic asymmetry and NCLBP

Significant between-group differences in PTAR, QAR, ADAR and PDAR were observed, which may suggest that asymmetry parameters relate to the occurrence of NCLBP in young adults. The present study did not observe a significant difference in pelvic asymmetry parameters in the frontal plane (AHAR and PHAR) between the two cohorts. This result suggests that asymmetry in the frontal plane is unlikely to be associated with the occurrence of NCLBP. These findings are supported by Levangie [4], who also reported pelvic asymmetry in the frontal plane was not positively related to the occurrence of NCLBP.

The logistic regression model suggested that BMI was significantly related to the NCLBP occurrence, which was supported by the findings of previous studies [34–36] that suggested overweight and high BMI increase tissue stress around the lumbar spine [34]. However, the finding that BMI was a factor associated with NCLBP occurrence must be interpreted with caution. This is because of the small actual difference in BMI observed between the NCLBP and the control group, and no participants in either group had the BMI beyond “normal” BMI classification. Despite published literature that reported association between BMI and NCLBP, the absolute difference in BMI was often very small, ranging between 0.59 [37] and 1.5 [38]. The small differences in BMI cast some doubts on the relationship between BMI and NCLBP.

Several studies have reported that anatomical variations of the lumbosacral and sacroiliac joints may also lead to pelvic asymmetries [39, 40]. The results of logistic regression analysis also reported that PTAR and PDAR were significant factors for NCLBP. The asymmetrical biomechanics of the articular surface of the lumbosacral and sacroiliac joints may increase one-sided muscle activity and subsequently lead to the occurrence of CLBP [41]. Previous studies showed that anterior pelvic tilt angle was associated with LBP because an increased anterior pelvic tilt could increase the strain of soft tissue in the lumbar region [36, 42, 43]. The standard deviation of anterior pelvic tilt angle in the NCLBP group observed in this study was higher than the standard deviation of 4.697 previously reported [36]. The larger standard deviation observed in the present study is likely to be related to the smaller sample group. Existing studies that investigated the impact of unilateral and bilateral dominant sports on pelvic torsion (pelvic rotation in the sagittal plane) reported that people who participated in unilateral dominant sports had a greater prevalence of pelvic asymmetry than those who participated in bilateral dominant sports and non-athlete groups. Bussey [12] found that athletes who engaged in bilateral dominant activities (e.g., running and cycling) showed decreased pelvic asymmetry (including lateral pelvic tilt and pelvic torsion) and lower rates of low back pain than those who engaged in unilateral dominant activities (e.g., hockey). Early literature proposed that pelvic torsion caused positional changes to one or both innominate bones of the sacroiliac joint [4]. The alteration of an innominate position may increase the stress to surrounding soft tissues because it is less efficient to dissipate the force from caudal or cephalad directions [4]. The results of the logistic regression analysis indicated that only PTAR and PDAR were significant factors for the occurrence of NCLBP and that AHAR and PHAR were not significant factors. This finding is a novel finding of the present study since the existing study mostly focused on assessing pelvic asymmetry in predominantly lateral pelvic tilt or pelvic torsion calculated by both the height and the width of the ASIS and PSIS. Previous studies showed that the pelvic asymmetry ratio would increase when the participant experienced not only pelvic torsion but also lateral pelvic tilt [44]. These findings indicated that the ratio or difference in the height between the left and right ASIS and PSIS could not specifically detect the predominant type of pelvic asymmetry (lateral pelvic tilt or pelvic torsion) for mixed pelvic asymmetry. Therefore, pelvic asymmetry may better be assessed by methods, such as the one proposed by Gnat and Bialy [8], that take into consideration the angular measurement and the ratio of the pelvis size to detect pelvic asymmetry in the sagittal plane.

### Limitations

The findings of the study should be interpreted with caution due to the limitations. Although BMI was found to be associated with pelvic asymmetry, the between-group difference in BMI was small. However, the difference in BMI between healthy individuals and people with low back pain observed in this study was similar with published studies that investigated risk factor of low back pain [37, 38]. It is currently unclear if the small difference may be of clinical significance. The power of sample size was not calculated and is likely to contain type two error and contribute to the significant between-group difference in BMI. Despite the statistically significant association between PTAR (pelvic asymmetry in the sagittal plane) and PDAR (pelvic asymmetry in the transverse plane) and the occurrence of NCLBP, the small odds ratio of PTAR (1.17) and PDAR (1.21) limited the interpretation of asymmetry from these two parameters. These two significant asymmetry parameters may not be clinically meaningful, and their roles in NCLBP remains unclear. The present study adopted a cross-sectional design, therefore causality cannot be inferred. Due to the small association between asymmetry parameters and LBP observed in the present study, future longitudinal studies are recommended to confirm the association between pelvic asymmetry in the sagittal plane/pelvic asymmetry in the transverse plane and LBP occurrence. The reliability of the GPS was only tested in the healthy group. This was because that it could not ascertain if people with NCLBP would have reproducible posture when measured with the GPS. Thus the first step was to establish the reliability of the device in the healthy population. Future study is recommended to explore the reliability for GPS in the NCLBP group. The interpretation of the results may not be generalized to patients who age outside the 20 to 27 range. The GPS in the present study employed the skin markers on pelvic bones to assess the pelvic parameters. Even though previous study showed acceptable validity [23] of the photographic assessment systems, skin markers on pelvic bones seem to have an impact on the pelvic parameters. The future study should also investigate the GPS validity. The camera used in the present study only had a 2 megapixel lens, which was likely to increase the measurement errors. Future studies could utilize higher resolution lenses to assess body posture.

## Conclusion

The study demonstrated an acceptable level of reliability of the GPS to assess pelvic posture asymmetry. However, not all pelvic asymmetry variables demonstrated the same level of reliability. It is currently unclear whether the observed reliability may be sufficient to identify intervention-induced changes in the symmetry ratio. Despite the pelvic tilt angle asymmetry ratio in the sagittal plane and the distance between the PSIS and the midline in the frontal plane were significantly associated with the occurrence of NCLBP, however, the associations were small which suggested a limited potential role of pelvic asymmetry may play in NCLBP occurrence.

## Data Availability

The dataset supporting the conclusions of this article is available from the authors upon request.

## Abbreviations

ADAR: The distance between the ASIS and the midline asymmetry ratio
AHAR: The height of the ASIS from the platform asymmetry ratio
ASIS: Anterior superior iliac spine
BMI: Body mass index
GPS: Global Postural System
ICC: Intraclass correlation coefficient
LBP: Low back pain
MDD_95_: Minimal detectable difference
NCLBP: Non-specific chronic low back pain
NRS: Numerical rating scale
OR: Odds ratio
PDAR: The distance between the PSIS and the midline asymmetry ratio
PHAR: The height of the PSIS from the platform asymmetry ratio
PSIS: Posterior superior iliac spine
PTAR: The pelvic tilt angle asymmetry ratio in the sagittal plane
QAR: Q angle asymmetry ratio
SEM: Standard error of measurement
